# Conformational Analysis, Molecular Structure and Solid State Simulation of the Antiviral Drug Acyclovir (Zovirax) Using Density Functional Theory Methods

**DOI:** 10.3390/ph7060695

**Published:** 2014-06-06

**Authors:** Margarita Clara Alvarez-Ros, Mauricio Alcolea Palafox

**Affiliations:** Departamento de Química-Física I. Facultad de Ciencias Químicas, Universidad Complutense, Ciudad Universitaria, Madrid 28040, Spain; E-Mail: maralva32@yahoo.es

**Keywords:** acyclovir, 9-(2-hydroxyethoxymethyl) guanine, tautomer, antiviral drug, conformational analysis, Zovirax

## Abstract

The five tautomers of the drug acyclovir (ACV) were determined and optimised at the MP2 and B3LYP quantum chemical levels of theory. The stability of the tautomers was correlated with different parameters. On the most stable tautomer N1 was carried out a comprehensive conformational analysis, and the whole conformational parameters (R, β, Φ, φ_1, _φ_2_, φ_3_, φ_4_, φ_5_) were studied as well as the NBO Natural atomic charges. The calculations were carried out with full relaxation of all geometrical parameters. The search located at least 78 stable structures within 8.5 kcal/mol electronic energy range of the global minimum, and classified in two groups according to the positive or negative value of the torsional angle φ_1_. In the nitrogen atoms and in the O2' and O5' oxygen atoms of the most stable conformer appear a higher reactivity than in the natural nucleoside deoxyguanosine. The solid state was simulated through a dimer and tetramer forms and the structural parameters were compared with the X-ray crystal data available. Several general conclusions were emphasized.

## 1. Introduction

**Figure 1:**
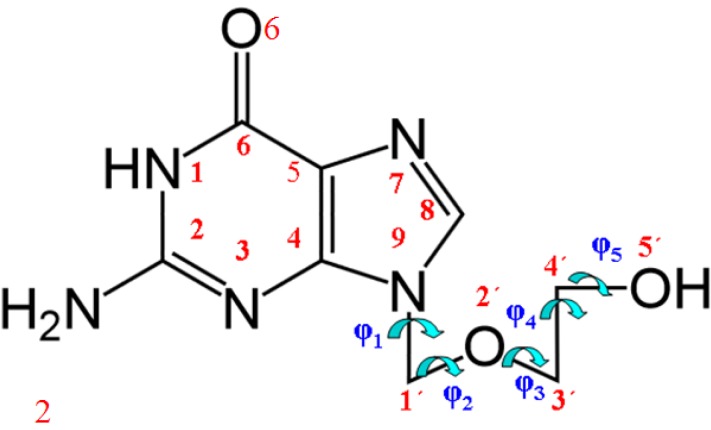
Molecular structure and definition of the torsional angles in tautomer N1 of acyclovir.

Acyclovir (ACV, 9-(2-hydroxyethoxymethyl) guanine, Zovirax [1,2], Figure 1), is a guanine derivative possessing antiviral activity and commonly used in the treatment of herpes. It is a potent antiviral agent that is used as a highly specific inhibitor of herpes viruses (HSV) types 1 and 2 [3,4,5,6,7]. A series of new guanine base modified tricyclic analogues of ACV and ganciclovir were evaluated for activity against herpes simplex virus type 1 and 2, showing similar antiherpetic potency as the parent compounds ACV and ganciclovir [8]. The antiherpetic activity was found to be strongly dependent on the nature and esteric demands of the substituents in the 6 and/or 7 positions [9].

Most persons who are infected with human immunodeficiency virus type (HIV-1) are also infected with herpes simplex type2 (HSV-2), which is associated with increased plasma and genital levels of HIV-1. Thus, it has been reported that ACV inhibits HIV upon human herpes virus (HHV) coinfection in tissue cultures. This activity was found to be correlated with the phosphorylation of the parent drug to the monophosphate form mediated by HHV-encoded kinase [10]. Recent studies show that ACV decreases the HIV-1 RN and suppress both viruses in coinfected tissues [11], with great impact on HIV and HSV-2 seropositive patients [12,13]. The treatment of ACV- resistant HSV infections [7,14] and tests for release it at oral therapeutic levels have been also reported [15].

As it is shown above, ACV has been extensively studied from the pharmaceutical and medical point of view. However, few studies appear on its molecular structure, only at low level [16], and to our knowledge, there is no data on its conformational characteristics by Density Functional Methods (DFT) or *ab initio* quantum chemical methods. Thus, it is one of the tasks of the present manuscript. Conformations of some derivatives of ACV with biological activities have been reported [17], as well as studies of complexes of ACV with several metals [18,19].

The tautomeric study of DNA structural components has a great interest today with numerous research publications [20,21]. The tautomeric equilibrium in ACV between the keto and enol forms has been observed from the UV/VIS spectra [22]. This equilibrium depends on the polarity of the solvent, and therefore, in water solution the keto form prevails, while in methylene chlorides it is the enol one. Thus, another goal of the present manuscript is to study the possible tautomers of ACV, and the determination of their % populations at room temperature and at 273.15 K. The stability of these tautomers and its dependence of different parameters is another point analyzed here.

Finally, from our understanding, would be interesting to calculate the different conformational possibilities of ACV and compare the results with the natural nucleoside deoxyguanosine (dG). This is the last goal of the present manuscript. An accurate knowledge of the different conformers of ACV, its charge distribution, inter- and intra-molecular interactions, solid structure, and flexibility would be an important help for the interpretation of drug-target interactions, as well as to design new antivirus.

For this reason, the conformers of natural and analogues nucleosides have been analyzed by different authors. Now, an extensive theoretical study of the conformational preferences in ACV has been carried out with full relaxation of all geometric parameters, in an attempt to gain insights into molecular features responsible for activity. We will attempt to determine herein, if the various geometric features in ACV are correlated or interact with one another. We are also interested in whether alternative forms of hydrogen bonding make significant contributions to the conformational behavior of ACV.

## 2. Computational Details

Calculations were carried out by using the Becke exchange functional (B) [23], Becke’s three-parameter exchange functional (B3) [24], Handy’s OPTX modification of Becke’s exchange functional (O) [25,26] and the extended (X3) [27], in combination with the correlation functionals of Lee, Yang, and Parr (LYP) [28], and Perdew and Wang’s 1991 (PW91) [29]. B3LYP Density Functional method (DFT) is the most used today, and for this reason the majority of the calculations were carried out with it.

All the methods appear implemented in the GAUSSIAN 03 program package [30]. The UNIX version with standard parameters of this package was used in the alpha computer of the Computational Centre from University Complutense of Madrid, in which all quantum chemical computations were performed. DFT methods provide adequate compromise between the desired accuracy and the heavy demands put on computer time and power. Different studies have shown that the data obtained with DFT methods are in good agreement with those obtained by expensive computational methods as MP2 [31,32,33,34,35]. Also they give good results in many studies on nucleosides and on drug design [36,37] and they predict vibrational frequencies of DNA bases better than HF and MP2 [38,39,40,41,42]. Moreover, because of the high size of ACV, MP2 calculations were only possible with the 6–31G(d) basis set for memory computer problems. However, we used the 6–31G(d,p) basis to calculate single point energies.

The 3D Potential Energy Surface (PES) of this molecule was determined by rotation of the exocyclic and endocyclic torsional angles φ_1_ (equivalent to χ angle in nucleosides [43]), φ_2_, φ_3_, φ_4_ and φ_5._ These dihedral angles were simultaneously hold fixed at values varying between 0° and 360° in steps of 60° in a first study. All other geometrical parameters were relaxed during these optimizations. 78 optimized geometries were obtained in this step by minimizing the energy with respect to all geometrical parameters without imposing molecular symmetry constraints. Berny optimizations under the TIGHT criterion were used. Atomic charges were determined with the Natural NBO procedure [44].

Harmonic wavenumber computations were carried out at the same level of the respective optimization process and by the analytic evaluation of the second derivative of the energy with respect to nuclear displacement. Vibrational frequencies calculations were performed on all optimized conformers to confirm that they corresponded to local minima. All optimized structures showed only positive harmonic vibrations (local energy minima). Relative energies were obtained by adding zero-point vibrational energies (ZPEs) to the total energy. For the calculation of the ZPEs, the frequencies were retained unscaled. The Δ*G* values were sums of electronic and thermal Free Energies. The conformational equilibrium at 298.15 K was evaluated by means of the Boltzmann distribution formula exp(−Δ*G*/kT), where Δ*G* is the relative Gibbs energy.

## 3. Results and Discussion

**Figure 2:**
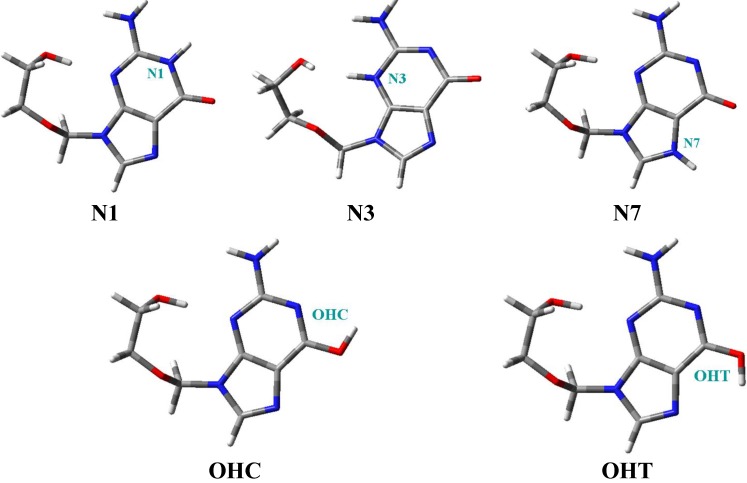
Five tautomers described in acyclovir.

ACV has five possible tautomers (Figure 2), that were fully optimized at different levels of computation, Table 1. The most stable one corresponds to N1, and thus we have focused the study only in this tautomer. The remaining forms were left for future research. In the last two column of this Table is shown the % population of the different tautomers at 298.15 K and 273.15 K. At room temperature the largest population corresponds to tautomer N1 (48.1%). The second population is due to OHC tautomer (37.7%) and the third one is to OHT (14.3%). Tautomers N3 and N7 have very little population, less than 0.05%.

**Table 1:** The optimum stable tautomers calculated in acyclovir molecule at the levels: B3LYP/6-311++G(3df,pd) (values in bold); MP2/6-31G(d,p) (values in key); B3LYP/6-31G(d,p) (values in normal); O3LYP/6-31G(d,p) (values in brackets); B3PW91/6-31G(d,p) (values in parenthesis); B3LYP/cc-pVDZ (values in quotation marks) and B972/6-31G(d,p) (values in italic type). Torsional angles in degrees, dipole moments in debyes, distance R in Å, energy increments in kcal·mol^−1^ and population (%) at 298.15 K (P_298.15_) and at 273.15 K (P_273.15_).

Tautomers	R	β	Φ	φ_1_	φ_2_	φ_3_	φ_4_	φ_5_	μ	ΔE	ΔG	P_298.15_	P_273.15_
**N1**	**3.963**	**72.5**	**54.3**	**−75.3**	**146.4**	**−88.5**	**69.8**	**−69.3**	**5.739**	**0** **^a^**			
	{3.892}	{73.5}	{51.3}	{−71.9}	{150.3}	{−94.6}	{69.1}	{−75.4}	{6.207}	{0} ^b^			
	3.925	72.3	53.7	−74.1	144.7	−93.4	71.1	−73.3	5.375	0 ^c^	0 ^d^	48.1	49.2
	[3.956]	[72.7]	[53.9]	[−75.4]	[144.5]	[−94.0]	[71.5]	[−72.1]	[5.369]	[0] ^e^	[0.638] ^f^		
	(3.891)	(72.7)	(53.2)	(−75.1)	(145.2)	(−92.3)	(70.3)	(−74.1)	(5.492)	(0) ^g^	(0) ^h^		
	“3.922”	“72.0”	“54.0”	“−73.9”	“143.2”	“−92.0”	“72.3”	“−74.4”	“5.34”	“0” ^i^	“0” ^j^		
	*3.913*	*72.8*	*53.3*	−*75.0*	*145.3*	−*93.4*	*70.8*	− *73.6*	5.502	*0* ^k^	*0* ^l^		
**N3**	{3.672}	{77.0}	{53.5}	{−45.9}	{149.1}	{−92.5}	{60.2}	{−92.6}	{12.220}	{25.457}			
	3.877	80.5	49.5	−48.4	155.0	−93.9	61.1	−88.	11.183	25.529	24.811	<0.05	<0.05
**N7**	{3.741}	{73.0}	{52.0}	{−76.4}	{140.5}	{−80.8}	{72.4}	{−92.7}	{9.841}	{19.302}			
	3.745	72.8	53.3	−79.9	136.9	−78.5	71.8	−91.5	7.785	17.516	17.689	<0.05	<0.05
**OHC**	{3.826}	{73.5}	{51.7}	{−72.3}	{146.5}	{−89.2}	{70.4}	{−82.6}	{2.471}	{0.603}			
	3.839	72.9	53.4	−75.9	141.9	−87.0	71.1	−81.5	2.100	0.145	0.284	37.7	37.7
**OHT**	{3.792}	{73.5}	{52.0}	{−72.4}	{145.0}	{−87.2}	{70.6}	{−86.1}	{3.437}	{1.131}			
	3.806	72.9	53.6	−76.2	140.7	−85.0	71.0	−84.9	2.105	0.719	0.830	14.3	13.1

^a^ Δ*E* = 0 = −811.214731 a.u. at B3LYP/6-311++G(3df,pd) level; ^b^ Δ*E* = 0 = −808.643998 a.u. at MP2/6-31G(d,p) level; ^c^ Δ*E* = 0 = −810.723396 a.u. at B3LYP/6-31G(d,p) level; ^d^ Δ*G* = 0 = −810.764701 a.u.; ^e^ Δ*E* = 0 = −810.449499 a.u. at O3LYP/6-31G(d,p) level; ^f^ Δ*G* = 0 = −810.490782 a.u.; ^g^ Δ*E* = 0 = −810.419555 a.u. at B3PW91/6-31G(d,p) level; ^h^ Δ*G* = 0 = −810.460766 a.u.; ^i^ Δ*E* = 0 = −810.760402 a.u. at B3LYP/cc-pVDZ level; ^j^ Δ*G* = 0 = −810.801662 a.u.; ^k^ Δ*E* = 0 = −810.444324 a.u. at B972/6-31G(d,p) level; ^l^ Δ*G* = 0 = −810.485454 a.u.

**Figure 3:**
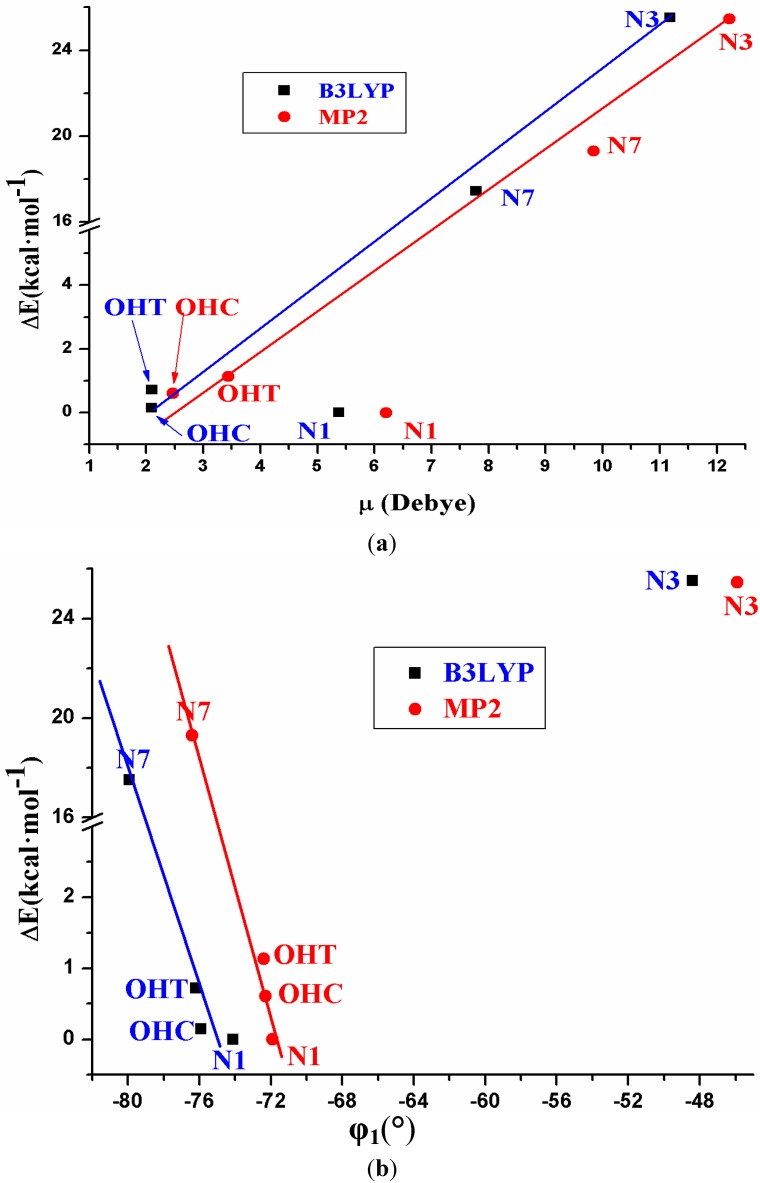
Relationship/tendency observed between the relative electronic energy Δ*E* + ZPE correction of the different tautomers *vs*. (**a**) the dipole moment μ. and (**b**) the exocyclic torsional angle φ_1_.

An analysis of the relative energies of these tautomers shows that they can be related to the dipole moment (μ) and to the torsional angle φ_1_, Figure 3. Thus, the least stable tautomer in the isolated state (N7) has the highest μ, *i.e.*, it is the most stable in water solution. Other relations can be observed between Δ*E* and φ_1_. It is noted that tautomer N7 has the highest negative value of φ_1_ angle.

### 3.1. Definition of the Conformational Angles

The atomic description of tautomer N1 of ACV, as well as the most important exocyclic and endocyclic torsional angles, is defined in Figure 1. The conformation can be characterized by the following five torsional angles: (i) the torsional angle, φ_1_(C4-N9-C1'-O2'), which determines the two orientations of the base relative to the opened chain, denoted in the present manuscript as conformers A and B; (ii) The exocyclic torsional angle φ_2_ (N9-C1'-O2'-C3'); (iii) φ_3_ (C1'-O2'-C3'-C4') that determine the different folds of the chain; (iv) φ_4 _(O2'-C3'-C4'-O5') describing the O5'H orientation; and (v) the torsional angle φ_5_ (C3'-C4'-O5'-H5') that define the orientation of the hydroxyl hydrogen H5'. This φ_5_ angle has some similarities with the β angle of the nucleosides [43].

Considering the structure of the chain, that likes the structure of the sugar in the nucleosides, and in accordance to previous works [45], other three structural parameters were defined to fix the chain position respect to the plane of the nucleobase: (vi) The vector R (N9···O5') which determines the distance of the OH group relative to the base; (vii) The angle β (C4-N9···O5') which defines the angle of the OH group relative to the base plane; and (viii) the angle Φ (C1'-N9···O5') which also determines the position of the OH group.

### 3.2. Conformers and Energetics

An extensive conformational study of tautomer N1 was carried out through a rotation of the exocyclic φ_1_, φ_2_, φ_3_, φ_4 _and φ_5_ torsional angles. A detailed collection of the most important conformational parameters of these optimized forms is included in Table 2. The conformers were classified according to the two ranges of rotation of φ_1_: conformers A with the φ_1 _values negative and conformers B with φ_1_ positive.

Two energy criteria were considered for each conformer: the electronic energy Δ*E* + ZPE correction, and the Gibbs energy Δ*G*. For the numbering of the conformers in each range of rotation of φ_1_ was followed the Δ*E* + ZPE criterion. Calculations at different levels, as well as single point calculations at the MP2/6-31G(d,p)//B3LYP/6-31G(d,p) level were carried out to confirm the stability of the main conformers, Table 2 and Table 3. In general, the stability order remains, although several changes are observed. Thus, conformer B2 appears as the most stable one instead of A1 predicted by the DFT methods. The % population of the different conformers at 298.15 K (P_298.15_) and at 273.15 K (P_273.15_) was calculated. It indicates that only confomers A1 (41.5%), B1 (36.1%), B2 (9.6%) and A2 (8.7%) have importance. The population of the remaining conformers is lower than 1% and thus they are not of interest. The temperature effect is not significant.

By the same methodology, the relative energies of the different conformers have been determined in related nucleosides [34,46,47,48]. The global minimum calculated in these molecules by MP2/6-31G(d,p) was in accordance to that found by B3LYP/6-31G(d,p). Thus, our results by B3LYP can be considered acceptable.

**Table 2:** The 78 optimum stable conformers calculated in tautomer N1 of acyclovir molecule at the levels: B3LYP/6-311++G(3df,pd) (values in bold); MP2/6-31G(d) (values in keys); B3LYP/6-31G(d,p) (values in normal type); O3LYP/6-31G(d,p) (values in brackets); B3PW91/6-31G(d,p) (values in parenthesis); B3LYP/cc-pVDZ (values in quotation marks) and B972/6-31G(d,p) (values in italic type) level. Distance R in Å, torsional angles in degrees, dipole moments in debyes, and energy increments in kcal·mol^−1^.

Conformers	R	β ^a^	Φ ^a^	φ_1_	φ_2_	φ_3_	φ_4_	φ_5_	μ	ΔE	ΔG
A1	**3.963**	**72.5**	**54.3**	**−75.3**	**146.4**	**−88.5**	**69.8**	**−69.3**	**5.739**	**0** **^b^**	
	{2.859}	{73.8}	{50.9}	{−71.8}	{151.8}	{−96.3}	{68.8}	{−74.2}	{6.182}	{0.064}	
	3.925	72.3	53.7	−74.1	144.7	−93.4	71.1	−73.3	5.375	0 ^c^	0 ^d^
	[3.956]	[72.7]	[53.9]	[−75.4]	[144.5]	[−94.0]	[71.5]	[−72.1]	[5.369]	[0] ^e^	[0.638]
	(3.891)	(72.7)	(53.2)	(−75.1)	(145.2)	(−92.3)	(70.3)	(−74.1)	(5.492)	(0) ^f^	(0) ^g^
	“3.922”	“72.0”	“54.0”	“−73.9”	“143.2”	“−92.0”	“72.3”	“−74.4”	“5.343”	“0” ^h^	“0” ^i^
	*3.913*	*72.8*	*53.3*	− *75.0*	*145.3*	− *93.4*	*70.8*	− *73.6*	*5.502*	*0* ^j^	*0* ^k^
A2	**3.774**	**76.8**	**52.5**	**−97.7**	**65.9**	**74.6**	**−67.0**	**−34.1**	**5.367**	**1.258**	
	{3.727}	{77.7}	{50.7}	{−96.9}	{66.0}	{76.2}	{−65.5}	{−42.7}	{5.776}	{0.095}	
	2.892	77.3	51.5	−97.0	67.7	72.4	−65.1	−39.3	5.382	0.928	1.324
	[3.779]	[77.9]	[51.3]	[−98.8]	[68.1]	[72.8]	[−65.5]	[−38.3]	[5.361]	[1.189]	[2.009]
	(3.715)	(77.0)	(51.6]	(−97.4)	(67.4)	(72.8)	(−65.4)	(−38.6)	(5.436)	(0.655)	(1.034)
	“3.730”	“77.1”	“51.6”	“−96.6”	“67.6”	“72.4”	“−64.9”	“−38.4”	“5.385”	“1.000”	“1.370”
	*3.736*	*77.4*	*51.5*	− *97.9*	*67.4*	*73.2*	− *65.5*	− *38.7*	*5.438*	*1.000*	*1.018*
A3	**5.155**	**118.5**	**37.5**	**−107.7**	**71.0**	**171.1**	**−63.7**	**57.6**	**5.762**	**1.202**	
	{5.004}	{111.4}	{37.6}	{−101.9}	{64.7}	{164.9}	{−61.1}	{53.9}	{5.586}	{2.240}	
	5.104	118.6	36.4	−108.5	69.9	168.3	−61.1	53.4	5.594	2.303	1.014
	[5.149]	[121.2]	[36.1]	[−112.1]	[71.9]	[168.7]	[−63.0]	[55.9]	[5.653]	[0.585]	[0.073]
	(5.079)	(119.1)	(36.8)	(−110.2)	(69.3)	(168.7)	(−60.9)	(52.4)	(5.702)	(1.795)	(0.553)
	“5.105”	“120.0”	“36.7”	“−109.9”	“71.0”	“169.9”	“−60.7”	“52.5”	“5.610”	“2.864”	“1.513”
	*5.094*	*119.1*	*36.7*	− *110.0*	*69.6*	*168.6*	− *61.9*	*54.8*	*5.673*	*2.864*	*0.095*
A4	**4.592**	**116.2**	**64.7**	**−106.0**	**73.2**	**178.6**	**65.0**	**−56.8**	**7.630**	**1.726**	
	{4.487}	{113.4}	{64.9}	{−100.7}	{67.1}	{178.0}	{61.3}	{−52.8}	{8.026}	{2.887}	
	4.517	117.5	65.1	−106.7	71.7	−179.5	62.2	−52.9	7.390	2.765	1.457
	[4.552]	[119.3	[65.0]	[−109.9]	[73.7]	[−179.3]	[64.3]	[−55.3]	[7.309]	[1.030]	[0.432]
	(4.500)	(118.5)	(65.1)	(−107.9)	(71.0)	(−179.6)	(62.0)	(−52.8)	(7.480)	(2.309)	(1.009)
	“4.500”	“117.5”	“65.1”	“−107”	“71.0”	“−179.6”	“62.0”	“−52.8”	“7.480”	“3.256”	“1.871”
	*4.515*	*118.4*	*65.1*	−*108.2*	*71.4*	−*179.7*	*62.9*	−*54.0*	*7.467*	*3.256*	*0.503*
A5	5.119	123.3	36.2	−81.4	−71.2	−170.1	60.7	−53.6	5.166	2.952	1.976
A6	4.839	130.3	50.2	−93.9	−178.3	174.3	−60.6	54.4	7.387	3.870	2.472
A7	4.342	110.7	69.0	−100.5	73.3	−114.2	−57.0	53.3	4.342	4.088	2.821
A8	5.314	99.1	61.8	−108.8	70.2	174.4	179.8	123.1	5.490	4.529	3.020
A9	4.316	72.3	29.9	−100.9	86.9	94.8	−69.7	165.3	8.401	4.540	3.284
A10	4.317	98.8	29.8	−100.4	87.1	62.4	118.4	−119.6	8.402	4.541	3.295
A11	4.316	98.8	29.8	−100.5	87.1	94.7	−69.8	165.6	8.403	4.541	3.293
A12	5.248	97.3	61.6	−107.1	71.8	176.2	−179.4	177.3	6.700	4.778	3.132
A13	5.137	115.6	37.5	−109.6	69.9	168.6	−72.0	169.7	6.500	4.912	3.329
A14	3.804	130.6	83.0	−137.1	68.2	−147.3	63.6	64.2	7.916	4.944	4.017
A15	3.851	140.4	75.7	−114.8	−115.6	140.7	−61.6	63.4	7.669	5.082	3.917
A16	4.561	120.1	66.8	−112.4	70.5	−178.3	63.9	60.5	7.527	5.103	3.411
A17	4.400	99.7	34.4	−112.0	67.2	57.8	44.9	48.3	6.225	5.139	4.217
A18	5.204	119.3	39.3	−110.7	69.8	173.8	−64.3	−63.3	7.512	5.216	3.730
A19	3.411	68.6	64.3	−92.1	98.3	−114.1	64.4	39.1	7.760	5.354	4.890
A20	4.387	136.6	10.3	−88.2	158.8	79.4	−71.0	172.6	8.042	5.378	3.969
A21	3.562	167.7	60.8	−94.2	−126.1	83.2	−74.5	178.0	6.300	5.384	4.494
A22	4.486	118.5	67.8	−112.8	70.8	−179.7	71.7	−171.9	8.081	5.432	3.764
A23	5.209	88.5	51.3	−104.5	77.3	91.3	180.0	−69.9	6.218	5.476	3.817
A24	4.381	115.3	11.5	−102.7	−162.7	−77.3	73.0	−170.7	8.685	5.514	4.179
A25	4.948	80.5	59.5	−102.5	67.2	74.6	176.3	−68.2	6.425	5.588	4.277
A26	4.655	80.0	78.1	−99.5	74.6	−104.0	−175.2	72.4	7.000	5.633	4.093
A27	4.609	83.1	78.2	−102.2	71.5	−107.6	−174.8	179.6	5.473	5.662	4.371
A28	5.146	128.0	36.4	−81.4	−70.5	−167.5	71.8	−170.7	6.229	5.675	4.396
A29	4.653	84.8	51.8	−103.7	70.9	−109.1	−174.5	−74.1	6.731	5.845	4.325
A30	5.235	90.1	49.7	−102.9	80.0	96.4	179.4	−173.8	7.848	5.850	4.085
A31	4.400	99.7	34.4	−112.0	67.2	57.8	44.9	48.3	6.225	5.890	4.508
A32	5.220	123.3	38.2	−81.4	−70.2	−172.6	64.6	64.8	4.345	5.909	4.708
A33	4.415	114.1	12.6	−104.1	−163.1	−74.6	69.3	69.4	7.384	5.917	4.782
A34	5.955	104.4	31.3	−95.4	179.0	−178.1	179.2	72.9	6.080	6.174	4.503
A35	5.898	124.0	30.3	−96.6	179.3	179.3	179.9	179.6	6.676	6.195	4.544
A36	5.956	124.3	31.3	−96.4	179.5	178.1	−179.0	−72.9	7.716	6.254	4.762
A37	4.565	102.4	34.8	−112.6	66.4	60.5	56.3	−178.8	7.684	6.290	5.085
A38	4.273	110.2	72.9	−105.8	71.1	−103.2	−66.6	167.8	6.458	6.432	4.855
A39	4.970	138.6	46.2	−100.0	−179.8	172.1	−72.6	166.4	8.071	6.610	4.755
A40	5.011	104.7	46.2	−97.2	−179.7	−176.1	72.9	−166.1	7.023	6.779	5.097
A41	5.030	104.4	48.4	−95.2	179.6	−177.9	64.3	61.5	8.220	7.100	5.519
A42	5.722	115.4	19.7	−99.3	−176.9	−84.1	−179.2	70.4	6.405	7.372	5.289
A43	5.350	87.6	45.5	−90.3	138.5	−88.9	−177.6	−70.3	7.007	7.483	7.510
A44	5.386	92.4	40.2	−91.6	146.1	−91.1	178.5	173.8	7.133	7.627	5.567
A45	5.699	118.8	18.3	−101.1	−173.8	−84.4	−178.6	176.1	7.994	7.665	5.672
A46	5.765	120.0	17.5	−101.1	−170.2	−80.2	−173.9	−69.9	6.680	7.684	5.636
A47	4.832	106.9	33.0	−107.0	−164.9	−72.0	−62.7	178.1	8.554	8.451	6.636
B1	**3.928**	**73.4**	**53.4**	**75.1**	**−146.7**	**95.5**	**−68.8**	**72.8**	**5.812**	**0.072**	
	{3.885}	{74.5}	{50.3}	{70.1}	{−151.5}	{94.2}	{−68.1}	{76.8}	{6.204}	{0.056}	
	3.880	73.4	52.8	73.5	−145.4	90.9	−69.3	76.7	6.746	0.083	0.151
	[3.927]	[73.6]	[53.1]	[74.4]	[−145.4]	[92.6]	[−69.8]	[74.6]	[5.485]	[0.124]	[0.822]
	(3.855)	(73.5)	(55.6)	(74.2)	(−145.7)	(90.5)	(−68.9)	(76.7)	(5.595)	(0.061)	(0.108)
	“3.870”	“73.3”	“51.1”	“73.1”	“−144.2”	“89.6”	“−70.0”	“77.7”	“5.374”	“0.099”	“0.195”
	*3.882*	*73.7*	*52.7*	*74.0*	−*145.8*	*91.5*	−*69.3*	*76.0*	*5.601*	*0.099*	*0.090*
B2	**3.770**	**77.2**	**52.0**	**97.0**	**−66.7**	**−74.1**	**66.6**	**36.1**	**5.348**	**1.216**	
	{3.726}	{17.9}	{50.4}	{95.6}	{−66.8}	{−75.8}	{65.5}	{44.0}	{5.664}	{0}^l^	
	2.905	77.6	51.2	96.4	−68.5	−72.0	65.1	40.6	5.340	0.87	1.251
	[3.784]	[78.0]	[50.8]	[98.5]	[−68.9]	[−72.6]	[65.4]	[40.4]	[5.321]	[1.118]	[1.900]
	(3.713)	(77.3)	(51.2)	(96.8)	(−68.2)	(−72.4)	(65.4)	(39.9)	(5.404)	(0.612)	(0.975)
	“3.727”	“77.5”	“51.2”	“96.2”	“−68.4”	“−72.9”	“64.0”	“39.8”	“5.250”	“0.968”	“1.316”
	*3.736*	*77.8*	*51.1*	*97.3*	−*68.2*	−*72.8*	*65.4*	*40.3*	*5.398*	*0.968*	*0.941*
B3	**5.157**	**117.3**	**37.3**	**106.1**	**−71.7**	**−171.1**	**63.5**	**−57.6**	**5.951**	**1.157**	
	{5.007}	{110.9}	{37.4}	{100.4}	{−65.2}	{−165.1}	{60.9}	{−53.8}	{5.998}	{2.179}	
	5.104	117.9	36.2	107.1	−70.3	−168.1	60.9	−53.3	5.839	2.257	0.903
	[5.149]	[120.9]	[35.9]	[111.5]	[−72.1]	[−168.6]	[62.9]	[−55.9]	[5.872]	[0.542]	[0]^m^
	(5.080)	(118.1)	(36.5)	(108.2)	(−69.7)	(−168.2)	(60.8)	(−53.3)	(5.921)	(1.748)	(0.412)
	“5.106”	“119.5”	“36.5”	“108.6”	“−71.3”	“−169.7”	“60.6”	“−52.4”	“5.814”	“2.819”	“1.394”
	*5.094*	*118.6*	*36.6*	*108.9*	−*69.9*	−*168.4*	*61.7*	−*54.7*	*5.907*	*2.819*	*0.001*
B4	**4.529**	**95.5**	**65.4**	**78.3**	**70.8**	**−178.7**	**63.6**	**−54.4**	**6.470**	**2.031**	
	{3.673}	{66.1}	{78.5}	{56.6}	{63.8}	{−147.2}	{63.0}	{−52.4}	{6.649}	{1.707}	
	3.817	68.7	79.7	56.7	64.6	−149.1	63.8	−50.2	5.870	2.722	2.366
	[4.300]	[85.9]	[70.0]	[69.9]	[70.9]	[−168.2]	[63.2]	[−52.1]	[6.144]	[1.331]	[0.867]
	(3.814)	(68.9)	(79.4)	(56.4)	(64.5)	(−150.0)	(63.4)	(−48.6)	(5.913)	(2.523)	(2.080)
	“3.839”	“68.8”	“79.2”	“55.9”	“65.4”	“−150.2”	“63.6”	“−48.6”	“5.680”	“3.179”	“2.791”
	*3.858*	*70.3*	*78.6*	*58.0*	*64.6*	−*151.4*	*63.4*	−*49.6*	*5.949*	*3.179*	*1.557*
B5	**4.598**	**115.9**	**64.6**	**105.4**	**−72.8**	**−178.2**	**−64.9**	**56.7**	**7.911**	**1.693**	
	{4.507}	{112.9}	{64.3}	{99.6}	{−66.5}	{−177.0}	{−61.1}	{52.8}	{8.520}	{2.832}	
	4.526	117.5	64.8	106.4	−71.4	180.0	−62.0	52.8	7.735	2.757	1.463
	[4.559]	[119.2]	[64.9]	[109.6]	[−73.3]	[179.7]	[−64.2]	[55.2]	[7.629]	[1.012]	[0.420]
	(4.510)	(118.4)	(64.8)	(107.6)	(−70.7)	−179.9	(−61.8)	(52.7)	(7.826)	(2.289)	(0.978)
	“4.524”	“117.6”	“64.7”	“106.6”	“−72.3”	“−179.9”	“−61.7”	“52.1”	“7.517”	“3.262”	“1.920”
	*4.524*	*118.3*	*64.8*	*107.6*	−*71.1*	−*179.8*	−*62.7*	*53.8*	*7.793*	*3.262*	*0.470*
B6	5.119	123.1	36.3	81.0	70.9	170.1	−60.7	54.1	5.460	2.993	2.035
B7	4.991	113.3	29.3	107.2	−74.7	−87.7	−56.9	46.5	6.027	3.636	2.427
B8	3.582	165.9	56.3	82.7	65.4	73.0	−75.2	178.4	6.463	4.240	3.708
B9	3.793	70.4	79.1	57.2	68.0	−93.9	−49.8	60.8	5.069	4.263	3.900
B10	4.325	99.2	29.1	98.3	−88.0	−94.7	69.5	−166.2	8.268	4.496	3.334
B11	5.318	99.2	61.7	108.3	−70.8	−175.3	179.6	71.1	5.640	4.508	3.006
B12	5.002	132.0	29.1	77.5	74.7	88.4	57.0	−46.2	5.222	4.791	3.824
B13	5.138	115.0	37.3	108.2	−70.2	−168.4	71.8	−170.5	6.133	4.845	3.212
B14	5.246	98.4	61.7	108.6	−71.5	−176.6	179.2	177.1	6.553	4.852	3.244
B15	5.312	99.2	62.1	108.6	−70.7	−175.8	−178.5	−70.6	7.964	4.893	3.413
B16	4.407	99.6	33.7	110.2	−68.1	−57.9	−44.6	−48.7	5.939	5.042	4.065
B17	5.205	118.2	39.0	109.3	−69.6	−172.9	64.6	63.9	7.429	5.101	3.551
B18	3.562	167.3	60.9	93.8	125.8	−83.0	74.6	−178.0	6.069	5.456	4.582
B19	5.277	89.9	49.5	103.3	−79.4	−94.8	−179.7	69.2	6.054	5.577	3.960
B20	4.876	78.3	61.1	101.0	−65.9	−75.2	179.3	−69.0	6.102	5.694	4.494
B21	5.253	91.0	49.4	103.8	−80.2	−97.6	−178.6	174.8	8.034	5.950	3.922
B22	4.534	101.8	35.0	112.6	−66.2	−59.3	−55.7	179.6	7.559	6.169	5.032
B23	3.146	83.0	95.1	73.1	67.3	−116.9	63.5	64.3	5.334	6.305	6.199
B24	4.610	77.9	77.5	79.0	72.8	−102.2	−173.9	−175.2	5.317	6.617	5.315
B25	4.365	150.0	33.5	76.4	67.0	59.5	40.5	41.6	5.882	6.677	5.427
B26	4.618	108.0	52.5	77.2	68.5	−105.1	−173.1	−79.3	5.308	6.689	5.500
B27	5.184	143.6	51.8	78.9	76.5	89.5	−178.9	−69.4	6.563	6.718	5.374
B28	4.635	114.4	78.3	79.8	73.8	−101.1	−174.8	72.3	7.031	6.831	5.483
B29	5.194	141.1	52.7	77.5	74.5	87.3	175.3	68.0	5.203	6.997	5.596
B30	5.129	142.9	52.3	79.1	76.5	90.0	−179.5	−175.2	7.031	7.015	5.609
B31	4.964	136.4	32.2	77.2	71.0	78.8	64.1	−178.9	5.033	7.508	6.051

^a^ Notation used from ref. [49]; ^b^ Δ*E* = 0 = −811.214731 a.u. at B3LYP/6-311++G(3df,pd) level; ^c^ Δ*E* = 0 = −810.723396 a.u. at B3LYP/6-31G(d,p) level; ^d^ Δ*G* = 0 = −810.764701 a.u.;^e^ Δ*E* = 0 = −810.449499 a.u. at O3LYP/6-31G(d,p) level; ^f^ Δ*E* = 0 = −810.419555 a.u. at B3LYP/cc-pVDZ level; ^g^ Δ*G* = 0 = −810.460766 a.u.; ^h^ Δ*E* = 0 = −810.760402 a.u. at B3PW91/6-31G(d,p) level; ^i^ Δ*G* = 0 = −810.801662 a.u.; ^j^ Δ*E* = 0 = −810.444324 a.u. at B972/6-31G(d,p) level; ^k^ Δ*G* = 0 = −810.485454 a.u.; ^l^ Δ*E* = 0 = −808.643998 a.u. at MP2/6-31G(d) level; ^m^ Δ*G* = 0 = −810.491798 a.u.

**Table 3:** Single point calculations at the MP2/6-31G(d,p)//B3LYP/6-31G(d,p) level.

Conformer	Δ*E*/kcal·mol^−1^
A1	0.143
A2	0.082
A3	2.553
A4	3.112
B1	0.122
B2	0
B3	2.491
B4	1.962
B5	3.071

The conformers differ in general very little in energy. Thus, in our calculations 78 optimized conformers were found within the electronic energy range Δ*E* = 0–8.5 kcal/mol, and Gibbs energy range Δ*G* = 0–6.6 kcal/mol with respect to the global minimum. This range of values of Δ*G* is smaller than that calculated in dG, in dT [50], 0–7 kcal/mol and in dU [31], 0–9 kcal/mol.

Only four conformers are found within the electronic energy range Δ*E* = 0–1.0 kcal/mol (by criterium of Δ*E* + ZPE), Table 2, with φ_1_ −72°/−97° as *g*^−^ and 70°/96° as *g*^+^ by MP2. Among these conformers, B1 has the highest dipole moment 6.20 D although very close to that in A1, 6.18 D. These conformers are slightly favored in a polarizable environment with water.

Another seven conformers appear within the electronic energy range Δ*E* = 1.0–3.0 kcal/mol, three values were *anti* (φ_1_ *ca.* −100°) and four *syn* (φ_1_ *ca.* 90°). The *anti* structures are the expected forms in the natural nucleosides that form the nucleotides and polynucleotides in biological systems [33,35,46,47,48]. The ratio *anti/syn* in conformers A is 0.8 in the low-energy group (<3 kcal/mol) but it increases up to 1.7 in the 3.0–8.5 kcal/mol range.

**Figure 4:**
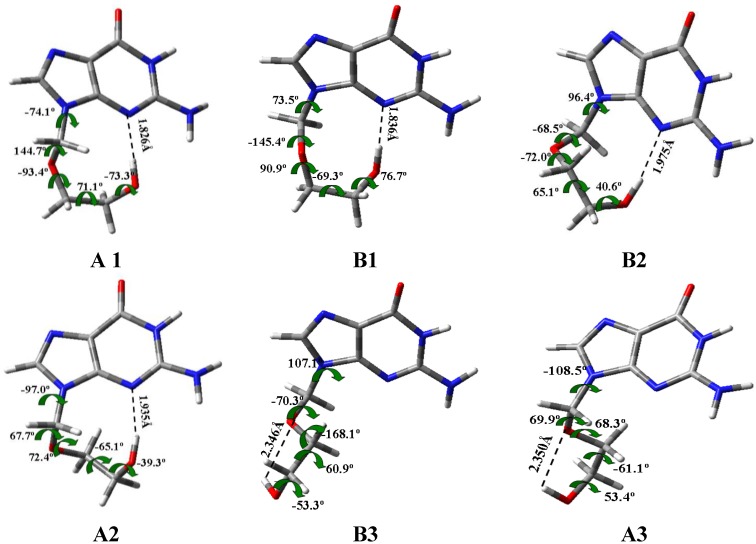
Geometry of the six most optimum conformers selected for each rotation angle φ_1_ determined in A1 conformer of N1 tautomer of acyclovir at B3LYP/6-31G(d,p) level. The values of the strongest intramolecular H-bonds are also included.

**Figure 5:**
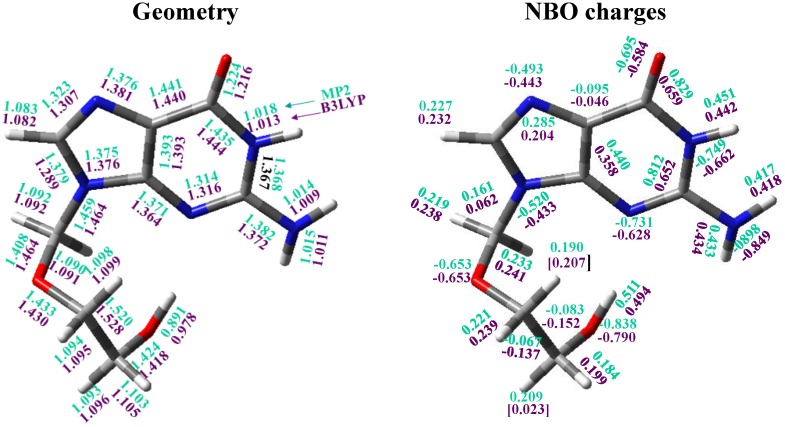
Natural atomic charges and optimum bond lengths in conformer B2 of tautomer N1 of acyclovir at B3LYP/6-31G(d,p) and MP2/6-31G(d,p) levels, in fuchsia and green colours, respectively.

The global minimum by DFT methods corresponds to the conformer denoted as A1 (Figure 4) and it appears stabilized by an intramolecular H-bond. The optimised bond lengths and natural NBO atomic charges on this conformer are collected in Figure 5. This global minimum by criterium of Δ*E* + ZPE agree well to that obtained by criterion of Δ*G,* but differs of that obtained by MP2/6-31G(d) and MP2/6-31G(d,p)//B3LYP/6-31G(d,p), Table 3 and Figure S3. It is because of the small difference in energy between both forms A1 and B2**.** This global minimum in the *syn* form by MP2 is in accordance to that obtained in other nucleosides [33,34,35] but differs of the *anti* form expected for the natural nucleosides and nucleotides in biological systems [43]. The second most stable conformer is B1 with values of φ_1_ = 70°, φ_2_ = −152° and φ_3_ = 94° by MP2. 

**Figure 6:**
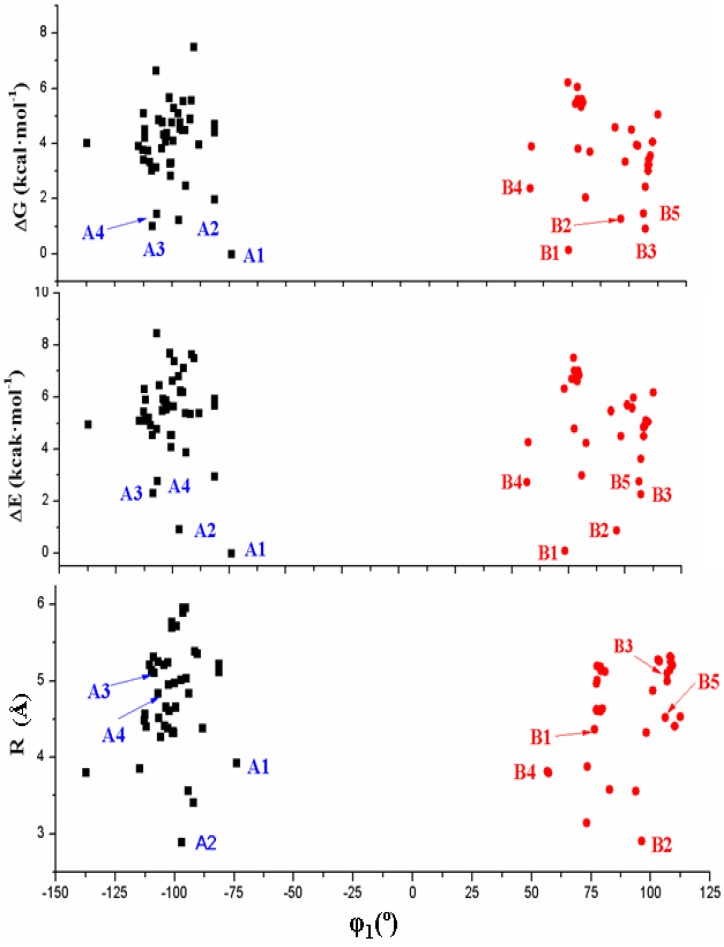
Distribution of the 78 optimum stable calculated conformers at the B3LYP/6-31G(d,p) level in tautomer N1 of acyclovir according to their exocyclic torsional angle φ_1_ and their: (**a**) relative electronic energy Δ*E* + ZPE correction; (**b**) relative Gibbs energy Δ*G*; and (**c**) the distance R. The most stable conformers of each type are pointed.

**Figure 7:**
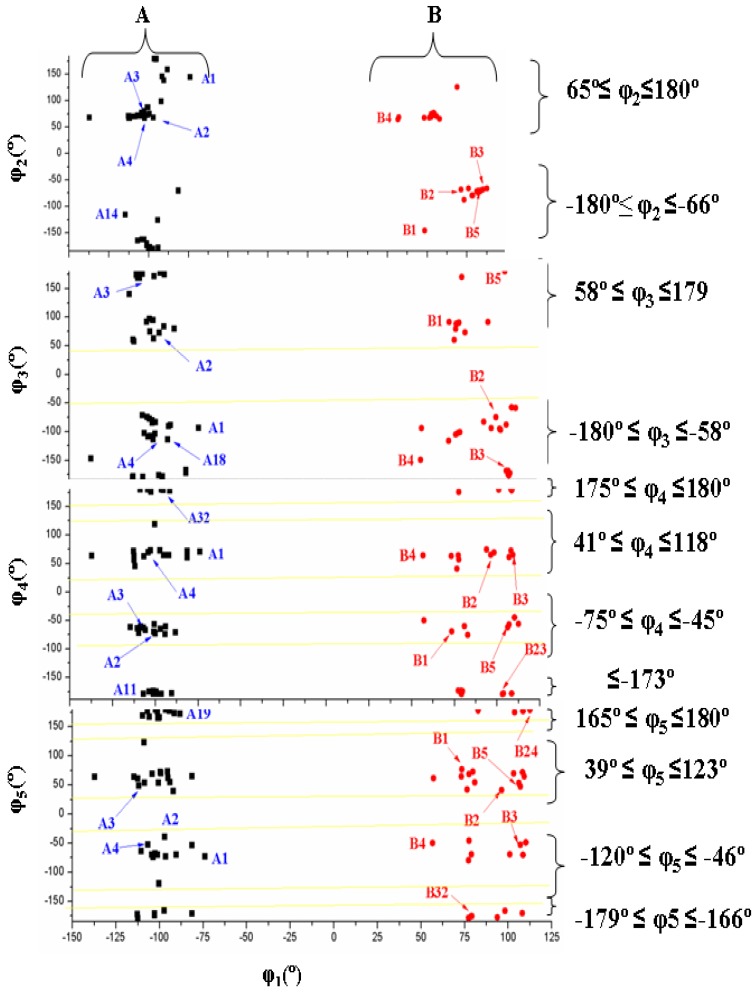
Distribution of the 78 optimum stable calculated conformers in tautomer N1 of acyclovir, according to the values of the exocyclic torsional angles: φ_2_. φ_3_. φ_4_ and φ_5_ versus the angle φ_1_.

**Figure 8:**
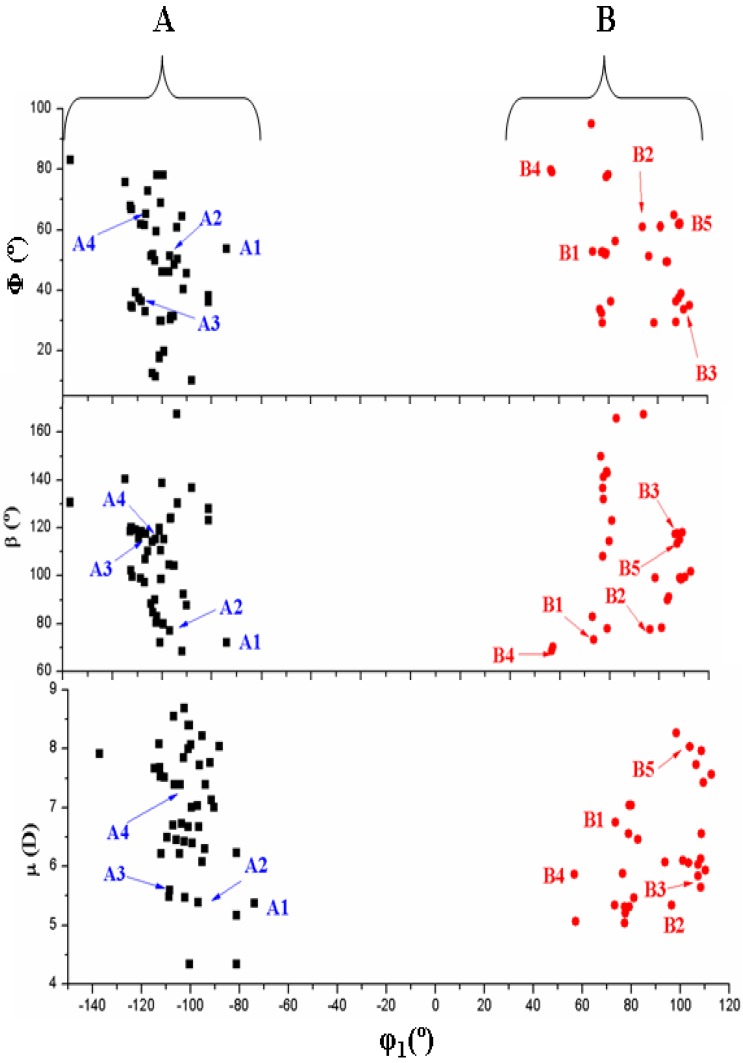
Distribution of the 78 optimum stable calculated conformers in tautomer N1 of acyclovir according to the values of the angles Φ and β, and the dipole moment μ versus the angle φ_1_.

**Figure 9:**
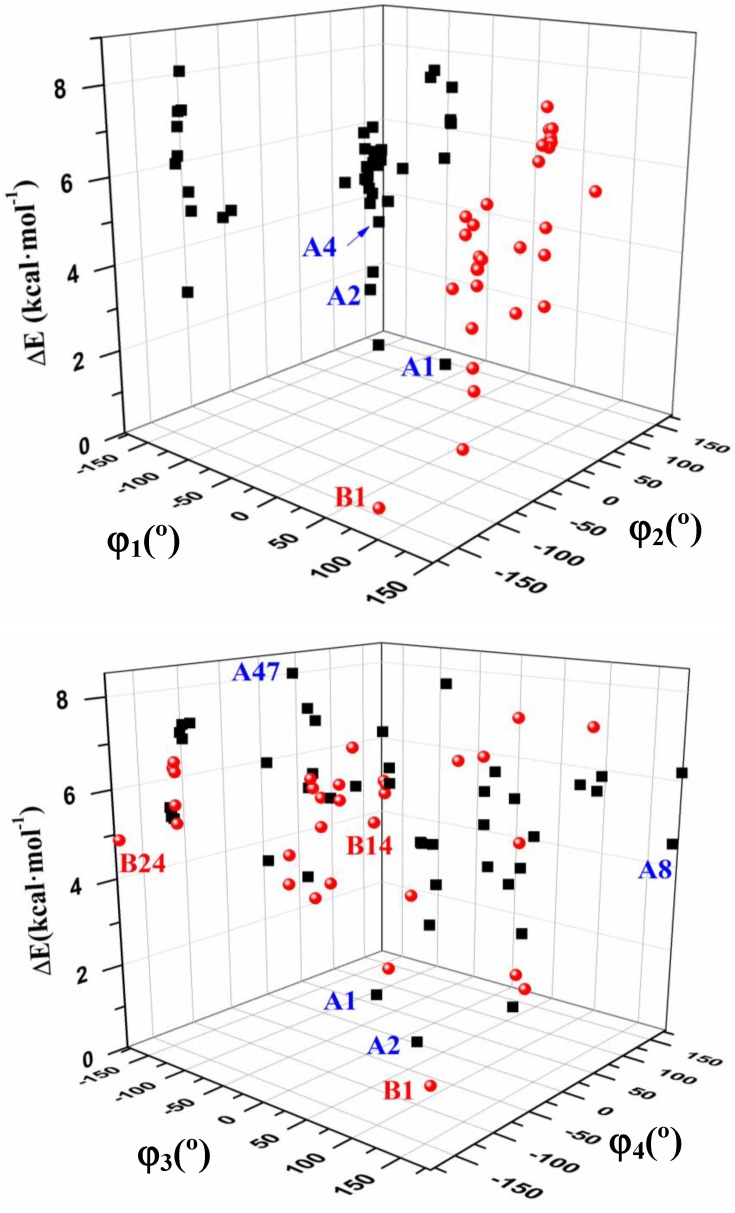
3D plots with the relative energies of the 78 optimum stable conformers according to the values of the exocyclic torsional angles: φ_1_. φ_2_. φ_3_ and φ_4_ versus relative electronic energy Δ*E* + ZPE.

Figure 4 shows the six best optimum conformers selected in the two ranges of φ_1_: three are **A** (A1–A3), and three are **B** (B1–B3). The values of the intramolecular H-bonds and the most important structural angles of each conformer are also included. Figure 6, Figure 7, Figure 8 and Figure 9 show the distribution of the 78 optimised conformers according to their energies, exocyclic torsional angles φ_1_–φ_5_, the angles β, Φ, and the vector R. The best significant conformers are pointed in these figures.

### 3.3. Conformational Angle Analysis

An overall examination of the five exocyclic torsional angles and two bond angles, defining the conformational space in tautomer N1 of ACV, leads to conclude the following, Figure 7, Figure 8 and Figure 9:

(i) The dihedral angle φ_1_ presents a bimodal distribution: (−137° ≤ −74°) 47 conformers **A** (*anti*) and (57° ≤ 113°) 31 conformers B (*syn*)**.** *Anti* forms prevail in number over *syn* ones and cover a slight wider range of φ_1_ values. This *anti* orientation has been found in the crystal of several nucleosides [51,52,53], and it is the form for biological activity [54]. Although, in many nucleoside analogues *syn* and *anti* forms have similar energy, however, the global minimum corresponds in general to the *syn* form, as in AZT [35]. In purine nucleosides there is relatively little restraint to rotation about the N9-C1' bond [55]. In ACV the high population of the *anti* forms and the wider range of φ_1_ values are factors that facilitate its antiviral activity. The sugar ring of the natural nucleoside dG can be mimics by the similar exocyclic angles of ACV
(ii) The torsional φ_2_ angle has a clear bimodal distribution: 65° ≤ φ_2_ ≤ 180° (49 conformers, 34 are A and 15 are B) and –180° ≤ φ_2 _≤ −66° (29 conformers, 13 are A and 16 are B), Figure 7
(iii) The φ_3_ angle also has a bimodal conformation: 58° ≤ φ_3 _≤ 180° (32 conformers, 22 are A and 10 are B) and −180° ≤ φ_3 _≤ −58° (46 conformers, 25 are A and 21 are B)
(iv) The φ_4_ angle has a trimodal distribution: −173° ≤ φ_4 _≤ 175° (28 conformers, 16 are A and 12 are B), 41° ≤ φ_4 _≤ 118° (28 conformers, 17 are A and 11 are B), and −75° ≤ φ_4 _≤ −45° (22 conformers, 14 are A and 8 are B)
(v) The torsional φ_5_ angle also has a trimodal distribution: −166° ≤ φ_5 _≤ 165° (29 conformers, 19 A and 10 B), 39° ≤ φ_5 _≤ 123° range (29 conformers, 16 A and 13 B), and −120° ≤ φ_5 _≤ −46° (20 conformers, 12 A and 8 B)
(vi) In the bond angle β has been observed a regular distribution in the 69° ≤ β ≤ 168° range, Figure 8. This large range indicates the high flexibility of the chain with multiple O5'H orientations. The most stable conformers with Δ*E* < 1 kcal·mol^−1^ have the β angle with values between 72° and 78°, while the conformers with higher Δ*E* have some values larger than 140°.
(vii) The bond Φ angle also shows a regular distribution in the 10° ≤ Φ ≤ 83° range for conformers A, and 29° ≤ Φ ≤ 95° for conformers B. The value of this angle appears between 51° and 54° for the four best conformers with Δ*E* < 1 kcal/mol^−1^. In general, the most stable conformers do not have large values of Φ, neither low values.

Finally, the R vector has a large range of values, between 2.905Å and 5.956 Å, and it determines the distance of the OH end of the chain from the base plane. 

### 3.4. Guanine Moiety

In the analysis of the six most stable conformers, the base heterocycle has a very small non-planarity, in general with torsional angles lower than 1°, with the exception of those involving N3 and N9 atoms with a value higher than 2°. It is due to the strong intramolecular H5'··N3 hydrogen bond which produces a ring deformation around N3. Thus, among the dihedral angles describing the base heterocycle nonplanarity, we have defined: ν_0_ (N1-C2=N3-C4), ν_1_ (C2=N3-C4=C5), ν_2_ (N3-C4=C5-C6), ν_3_ (N7=C8-N9-C4) and ν_4_ (C6-N1-C2=N3). 

An analysis of the six most stable conformers (Figure 4) shows that the highest base heterocycle nonplanarity corresponds to conformer A2 with the torsional angles ν_1_ (2.8°), ν_2_ (−2.3°) and ν_0_ (−1.3°). The other conformers show deviations higher than 1° in the following torsional angles: Conformer A1 in ν_2_ (−1.2°) and ν_3_ (−1.1°), conformer A3 in ν_1_ (1.5°) and ν_2 _(−1.3°), conformer B1 in ν_0_ (1.3°) and ν_1_ (1.1°), and conformer B2 in ν_4_ (1.2°).

Comparing the bond lengths of ACV (conformer A1) with those of dG, it is observed that ACV has several bonds of the guanine moiety larger than the corresponding bonds in dG, while in the remaining bonds are smaller. The largest difference appears in C4-N3.

### 3.5. The Side Chain

The most important characteristic of the structure of ACV is the conformation of the side chain that is attached to N9, which is characterized by the exocyclic torsional angles φ_1 _ to φ_5_, by the bond angles β and Φ that determine the position of the chain from the base, and by the distance R. The bonds along this chain can be in *trans* respect to each other and thus it gives an extended zig-zag structure or, by contrast, one or several alternate bonds can be in *gauche* in such a way that it resembles at least partially a portion of the furanose moiety of dG [49,56]. In this last case, it is capable of adopting conformations resembling a portion of the pentose ring, a factor which undoubtedly plays an important role in their biological activities [57]. Thus, three types of structures appear: two that have the side chain partially folded (conformers A and B), and one structure which has all bonds in *trans* orientation and leading to an almost planar zig-zag arrangement [49].

### 3.6. Intramolecular H-Bonds

Several authors have studied the intramolecular H-bonds in related nucleosides, in special using AIM method [58,59]. However, the absence of the furanose ring in ACV reduces largely the number of possible H-bonds. Therefore, only two intramolecular H-bonds may be observed in the main conformers of ACV: (i) The hydroxyl hydrogen H5'(O5') and the guanine’s position 3 nitrogen atom, H5'···N3, and (ii) The hydroxyl hydrogen H5'(O5') and the guanine’s position 2 oxygen atom, H5'···O2'.

Figure 4 shows these types of H-bonds in the six most stable conformers. H-bond (i) appears in conformers A1, B1, B2 and A2; while (ii) is observed in conformers B3 and A3 with values of 2.346 Å and 2.350 Å, respectively. H-bond (i) is stronger than (ii), and it gives a great stability to the structure. Thus, the conformers with the H-bond (i) are the most stable, Δ*E* < 1 kcal·mol^−1^, while the conformers with the H-bond (ii) have higher energy, Δ*E* > 2 kcal·mol^−1^.

### 3.7. Natural NBO Atomic Charges

The calculated values in conformer A1 appear collected in Figure 5. The largest negative charge corresponds to N2 and O5' atoms, −0.90 and −0.84 *e*, respectively by MP2 (where *e* is the charge of an electron). The next atom with large negative charge is N1 (*ca.* −0.7 *e*). The value of this charge is slightly higher than in dG, −0.69 *e*. The main effect of the NH_2_ group is a remarkable increment in the positive charge on C2, 0.81 *e*, because of the high negative charge on the amino nitrogen, −0.90 *e*. Consequently, a noticeable increase of the negative charge on N3 is observed, −0.73 *e* (−0.70 *e* in dG). The electron-rich sites of the guanine moiety are involved in H-bonds, with N3 and O2' acting as single acceptors.

In the nitrogen N9 the negative charge (−0.52 *e*) is lower than on N1, N3 and N7, but slightly higher than in dG. It is because in ACV the bonding to the chain increases the negative charge on N9. The value of the charge on N7 is important because in the neutral form of anti-tumour platinum drugs, the platinum atom has a strong preference for nitrogen N7 rather than for oxygen atoms of the base for its coordination [51]. Also, the N7 position in DNA is the most open to attack. N1 position is also important because when deprotonation of the weakly acid ACV occurs, the metal binding site changes to N1, which is the formally deprotonated site [60]. Raman spectra of related nucleosides in H_2_O indicate that the site of deprotonation in basic solutions is N1, while the site of protonation in acidic solutions is N3 and N7, the same sites that in its phosphorylated form. These results are useful for identification and characterization of its structure in natural occurring biopolymers [61].

The negative charge on the oxygen atoms is high, −0.65 *e* on O2' and −0.69 *e* on O6. The values in O2' and O5' are slightly higher than in dG, by B3LYP −0.603 *e* and −0.761 *e*, respectively. By contrast, in O6 the negative charge is lower than in dG, −0.592 *e*.

C6 is the atom with the highest positive charge, 0.829 *e* by MP2, in concordance to the high negative charge on O6, *i.e.*, it is the most reactive. With a slightly lower value appears C2 (0.812 *e*), and with much lower values H5'(O5^'^), H1(N1), H2(N2) and H2'(N2). The remaining hydrogen atoms have much less positive charge and they are less reactive. 

### 3.8. Solid State Simulation

The structure of ACV (CSD code CEHTAK) in the solid state was determined by X-ray diffraction [53,60,61,62,63]. Birnbaum *et al*. [49,62] obtained the crystals that belong to monoclinic space with three independent molecules (A, B and C), together with two water molecules [49]. Molecules A and B have similar conformation while that of molecule C is different, Table 4. In molecules A and B the angle φ_1_and φ_2_ are in the preferred *g*^−^ form, but in molecule C φ_2_ is in *trans*. It means that molecules A and B showed a partially folded conformation of the side chain, while molecule C appears extended [62]. Molecules A and B correspond to conformer A5 in the isolated state, while molecule C is represented by conformer A34. Molecules A and B are *ca.* 3 kcal·mol^−1^ more stable than molecule C. The difference in energy between molecule A and B is small and it is due to the flexibility of the side chains.

**Table 4:** A comparison of the most important structural parameters in the dimer and tetramer forms calculated at the B3LYP/6-31G(d,p) level in acyclovir molecule with those in the crystal. The torsional angles are in degrees and R in Å.

Solid form	R	β	Φ	φ_1_	φ_2_	φ_3_	φ_4_	φ_5_
**Dimer**								
Molecule A	5.150	127.9	−82.1	−82.1	−70.4	−168.4	71.9	−168.8
Molecule B	5.148	127.9	−88.8	−88.8	−70.3	−168.1	71.8	−168.9
**Tetramer**								
Molecule A	5.148	128.6	−82.3	−82.3	−70.4	−167.3	72.5	−167.4
Molecule B	5.212	117.2	−80.1	−80.1	−76.3	176.8	64.2	−90.8
Molecule C	5.110	122.0	158.6	158.6	−71.7	−50.5	60.1	−53.8
Molecule D	4.313	150.6	−95.3	−95.3	−93.1	−100.2	70.6	−160.4
**X-Ray** [62]								
Molecule A0				−76.5	−76.9	173.2	60.6	
Molecule B				−74.4	−66.3	−176.2	73.5	
Molecule C				−90.5	−173.3	−171.9	−174.4	

**Figure 10:**
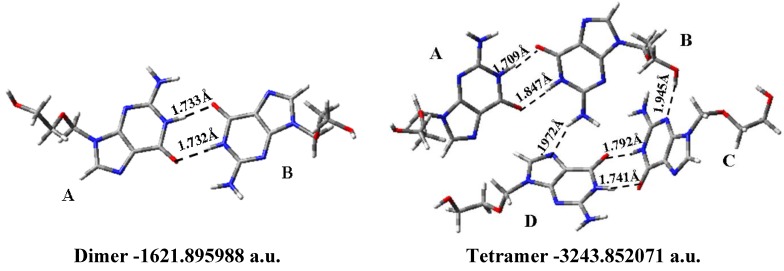
The optimized dimer and tetramer forms in conformer A1 of acyclovir at the B3LYP/6-31G(d,p) level. The H-bonds observed are in Å and the *E* (RB+HF-LYP) in a. u.

We have simulated this arrangement through a dimer and tetramer forms, Figure 10. The calculated value of the inter- and intramolecular H-bonds distances and the total energy is included in the Figure. The calculated bond lengths, bond angles and torsional angles are collected in Tables S1 and S2. The simulated structure is tightly bonded but not so tight as it the reported in the crystal. There are four donor sites: H1(N1), H2(N2), H2'(N2) and H5', and five possible acceptors: O6, N3, N7, O2' and O5'.

The dimer, and tetramer forms were built with the most stable conformer A1. In the dimer there are two intermolecular H-bonds between the two molecules: N1{in molecule A} and O6{in molecule B} 1.733 Å, and O6{in A} and N1{in B} 1.732 Å. The tetramer shows seven H-bonds: (i) two between molecule A and B: H_(N1)_{in molecule A} and O6{in molecule B} 1.709 Å, and O6{in molecule A} and H_(N1)_{in molecule B} 1.847 Å; (ii) There is only a H-bond between molecule B and C: H_(O5´)_{in B} and N3{in C}, 1.945 Å; (iii) There are two H-bonds between molecule C and D: H_(N1)_{in molecule C} and O6{in molecule D}, 1.741 Å, and O6{in C} and H_(N1)_{in D}, 1.792 Å. (iv) At list there are two H-bonds between molecule D and B: N7{in molecule D} and H_(N2__')_{in B}, 1.972 Å.

Comparing the calculated bond lengths and angles of molecule A in the dimer with the X-ray data [62], Tables S2 and S3, it is observed the good agreement in the values of our simulated structure. The greater differences appear in C4=C5 (0.025 Å), C2-N2 (0.023 Å), C3'-C4' and N1-C6 (0.016 Å), and the difference is almost null in C6=O, C5-N7, N7=C8 and C4-N9. A slight larger error is obtained when the comparison theory-experiment is carried out with the molecule B of the dimer. This error is slight reduced when the comparison is carried out with the simulated molecule A of the tetramer. In the angles the difference is almost null, with similar values of all the angles. Only several angles in the side chain show some noticeable differences, *i.e.*, C3'-C4'-O5', 4.7°.

Other four anhydrous forms of ACV and a new hydrate have been characterized by X-ray diffraction, with significant differences in the intermolecular H-bonding networks among the ACV forms [63,64,65]. A study of the solution forms of ACV shows that ACV can exist as polymorphic and pseudopolymorphic solvates [66]. We have simulated other dimer, trimer and pentamer forms with different conformers and collected in Figure S4.

## 4. Conclusions

In the present work we have shown a comprehensive compendium of the possible conformers in tautomer N1 of ACV. The geometries and values of the properties presented here appear to the most accurate to date. The most important findings of the present work are the following:

(1) Five tautomers of ACV were identified and fully optimized. At room temperature only tautomer N1 (48.1%), OHC (37.7%) and OHT (14.3%) have a noticeable population. It is very small in tautomers N3 and N7, less than 0.05%.
(2) The relative energies of the five tautomers appear related to the dipole moment and to the torsional angle φ_1_. The least stable tautomer N3 in the isolated state has the highest μ and thus, it is the most favoured in a polarisable environment with water.
(3) In the isolated state the most stable tautomer is N1 by both B3LYP and MP2 methods. In this tautomer, and through a rotation of φ_1_, φ_2_, φ_3_, φ_4_ and φ_5_ exocyclic torsional angles, 78 optimized stable conformers were identified, two *syn* and two *anti* failing into the 0–1 kcal·mol^−1^ Δ*E* + ZPE energy range.
(4) The calculated most stable conformer by all DFT levels corresponds to A1, while by MP2 is B2. In the nitrogen atoms and in the O2' and O5' oxygen atoms of conformer A1 appear a higher reactivity than in the corresponding natural nucleoside deoxyguanosine.
(5) The distribution of all the conformers according to the ranges of stability of the characteristic torsional angles was established. The values obtained indicate the flexible nature of ACV, which is higher than dG. An increase of the stability appears when the side chain is near to the purine base, with a value of R that fails into 3.925–2.892 Å range, and an angle Φ close to 54°.
(6) Only two intramolecular H-bonds may be observed in the main conformers of ACV, in contrast to the six H-bond types calculated in dG. It leads to a flexibility higher in ACV than in dG.
(7) The solid state was simulated through a dimer and tetramer forms. An excellent agreement with the X-ray crystal data was obtained, which indicates the good accuracy of the theoretical methods used.

## Supplementary Files

Supplementary File 1 Supplementary Material (DOC, 618 KB)
