# Supplementary material for: Conformational Analysis, Molecular Structure and Solid State Simulation of the Antiviral Drug Acyclovir (Zovirax) Using Density Functional Theory Methods
Source: Pharmaceuticals (Basel). 2014 Jun 6;7(6):695–722. doi: 10.3390/ph7060695 (PMC4078516; doi:10.3390/ph7060695)
Supplement: Supplementary File 1 — Supplementary Material (DOC, 618 KB) [file pharmaceuticals-07-00695-s001.doc]

Supplementary Material

**Table S1.** Bond lengths (in Å) of the dimer of ACV comparing to molecule A of X-ray [68].

| **Bond** | **Dimer** | | **X-ray [12]** |
| --- | --- | --- | --- |
|  | **A** | **B** | **A** |
| N1-C2 | 1.371 | 1.371 | 1.368 |
| N1-C6 | 1.404 | 1.404 | 1.388 |
| C2-N2´ | 1.359 | 1.358 | 1.336 |
| C2-N3 | 1.321 | 1.321 | 1.328 |
| N3-C4 | 1.350 | 1.350 | 1.348 |
| C4-C5 | 1.400 | 1.400 | 1.375 |
| C5-C6 | 1.429 | 1.429 | 1.419 |
| C6-O6 | 1.245 | 1.245 | 1.245 |
| C4-N9 | 1.377 | 1.377 | 1.376 |
| C5-N7 | 1.385 | 1.385 | 1.385 |
| N7-C8 | 1.305 | 1.305 | 1.304 |
| C8-N9 | 1.392 | 1.392 | 1.375 |
| N9-C1' | 1.457 | 1.457 | 1.462 |
| C1'-O2' | 1.399 | 1.399 | 1.408 |
| O2'-C3 | 1.423 | 1.423 | 1.436 |
| C3-C4' | 1.515 | 1.515 | 1.499 |
| C4'-O5' | 1.419 | 1.419 | 1.421 |

**Table S2.** Bond angles (in degrees) of the molecule A of the dimer and tetramer forms of ACV comparing to molecule A by X-ray [68].

| **Angle** | **Dimer A** | **Dimer B** | **Tetramer** | **X-ray** |
| --- | --- | --- | --- | --- |
| C2-N1-C6 | 125.7 | 125.7 | 125.6 | 125.6 |
| N1-C2-N2 | 115.7 | 115.7 | 115.6 | 117.1 |
| N1-C2-N3 | 123.9 | 123.9 | 124.0 | 123.2 |
| N2-C2-N3 | 120.4 | 120.4 | 120.3 | 119.7 |
| C2-N3-C4 | 112.1 | 112.1 | 112.1 | 112.3 |
| N3-C4-C5 | 129.0 | 129.0 | 128.8 | 128.7 |
| N3-C4-N9 | 125.7 | 125.7 | 126.0 | 125.8 |
| C5-C4-N9 | 105.3 | 105.3 | 105.2 | 105.5 |
| C4-C5-C6 | 118.0 | 118.0 | 118.2 | 118.6 |
| C4-C5-N7 | 111.0 | 111.0 | 110.9 | 110.9 |
| C6-C5-N7 | 131.0 | 131.0 | 130.8 | 130.5 |
| N1-C6-C5 | 111.3 | 111.3 | 111.2 | 111.7 |
| N1-C6-O6 | 119.6 | 119.6 | 120.0 | 120.1 |
| C5-C6-O6 | 129.1 | 129.1 | 128.8 | 128.3 |
| C5-N7-C8 | 104.2 | 104.2 | 104.6 | 104.3 |
| N7-C8-N9 | 113.7 | 105.8 | 113.2 | 113.1 |
| C4-N9-C8 | 105.8 | 105.8 | 106.1 | 106.2 |
| C4-N9-C1' | 126.2 | 126.2 | 126.1 | 125.7 |
| C8-N9-C1' | 128.0 | 128.0 | 127.6 | 128.0 |
| N9-C1'-O2' | 114.3 | 114.3 | 114.2 | 111.5 |
| C1'-O2'-C3' | 114.9 | 115.0 | 115.0 | 113.5 |
| O2'-C3'-C4' | 108.8 | 108.8 | 108.8 | 109.4 |
| C3'-C4'-O5' | 108.6 | 108.6 | 108.5 | 113.2 |

**Figure S1.** Natural atomic charges and optimum bond lengths in conformer A1 and other conformers of tautomer N1 of acyclovir at B3LYP/6-31G(d,p) and MP2/6-31G(d) (values in brackets) levels.

| **Geometry** | **NBO charges** |
| --- | --- |
| 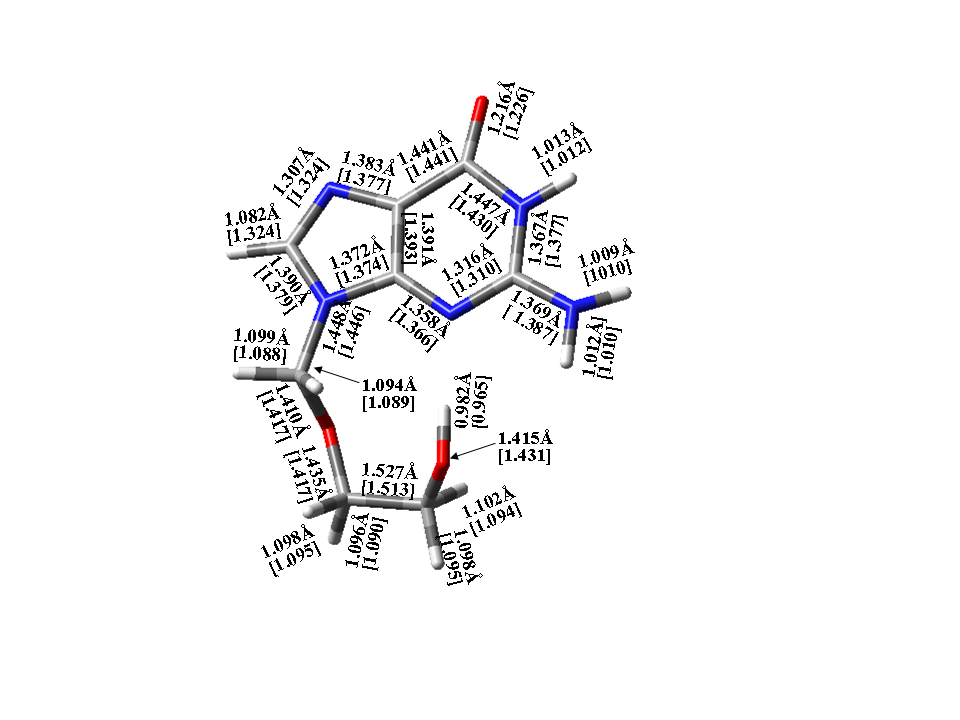 |  |

**Figure S2.** Other optimized dimer, trimer and pentamer forms of ACV.
